# Eligibility for the initiation of HIV treatment in the context of the updated EACS guidelines: results of a clinical audit

**DOI:** 10.1186/1758-2652-13-S4-P22

**Published:** 2010-11-08

**Authors:** MC Aichelburg, M Schlag, A Rieger

**Affiliations:** 1 Medical University of Vienna, Vienna, Austria; 2 Gilead Sciences, Vienna, Austria

## Background

Based on the recent update (November 2009) of the European AIDS Clinical Society (EACS) guidelines for HIV-1-infected patients, initiation of antiretroviral therapy (ART) is now also recommended in patients with 350 to 500 CD4+ T cells/µl and additional risk factors.

## Methods

In this single-center audit all ART-naïve HIV-1-infected subjects were screened for their eligibility to start ART based on CDC stage of disease, CD4+ T cell count and EACS-defined risk factors to assess the impact of implementing the new recommendations on clinical practice.

## Results

In January 2010, 155 out of a total of 994 HIV-1-infected subjects (16%) under regular observation at the HIV outpatient clinic of the Vienna General Hospital were ART-naïve.

47.7% (74/155) of individuals had at least one EACS-defined risk factor. Immediate initiation of ART was indicated in 10 subjects with symptomatic HIV-disease (6.5%). Of the remaining 145 asymptomatic patients, 15 (10.3%) had two consecutive CD4+ T cell counts < 350/µl and 19 subjects (13.1%) with a CD4+ T cell count 350-500/µl had ≥ 1 EACS-defined risk factor. Of the latter 19 subjects, six had HBV and/or HCV co-infection and one patient was suffering from HIV-associated nephropathy, whereas the remaining 12 subjects had other risk factors, e.g. age > 50 years, HIV-1 RNA level > 100000 copies/ml and/or CD4+ T cell decline > 100/µl within 1 year. Thus, according to the current EACS guidelines initiation of ART was recommended in 20% (31/155) and should be considered in additional 8% (12/155) of treatment-naïve patients. In contrast, treatment would have been recommended in only 16% of patients (25/155) based on the previous version of the EACS guidelines.

## Conclusions

In this clinical audit more than 1/4 of all treatment-naïve patients were eligible for the initiation of ART based on the updated EACS guidelines representing an increase of 76% over previous recommendations due to the inclusion of patients so far not considered eligible. Unless further studies show that ART initiation would be beneficial in all patients with a CD4+ T cell count <500/µl, awareness for HIV-associated and non HIV-risk factors needs to be increased to identify patients for whom treatment is recommended.

